# The cost-utility of point-of-care troponin testing to diagnose acute coronary syndrome in primary care

**DOI:** 10.1186/s12872-017-0647-6

**Published:** 2017-08-02

**Authors:** Michelle M. A. Kip, Hendrik Koffijberg, Marco J. Moesker, Maarten J. IJzerman, Ron Kusters

**Affiliations:** 1 Department of Health Technology and Services Research, MIRA institute for Biomedical Technology and Technical Medicine, University of Twente, Enschede, the Netherlands; 2 Laboratory for Clinical Chemistry and Haematology, Jeroen Bosch Ziekenhuis, Den Bosch, the Netherlands

**Keywords:** Cost-utility, Early health technology assessment, Point-of-care testing, Acute coronary syndrome

## Abstract

**Background:**

The added value of using a point-of-care (POC) troponin test in primary care to rule out acute coronary syndrome (ACS) is debated because test sensitivity is inadequate early after symptom onset. This study investigates the potential cost-utility of diagnosing ACS by a general practitioner (GP) when a POC troponin test is available versus GP assessment only.

**Methods:**

A patient-level simulation model was developed, representing a hypothetical cohort of the Dutch population (>35 years) consulting the GP with chest complaints. All health related consequences as well as cost consequences were included. Both symptom duration, selection of patients in whom the POC troponin test is performed, and test performance at different time points were incorporated. Health outcomes were expressed as Quality-Adjusted Life Years (QALYs). The main outcome parameters involve the effect of POC troponin testing on (in)correct hospital referrals, QALYs, and costs.

**Results:**

The POC troponin strategy decreases the referral rate in non-ACS patients from 38.46% to 31.85%. Despite a small increase in non-referral among ACS patients from 0.22% to 0.27%, the overall health effect is negligible. Costs will decrease with €77.25/patient (95% CI €-126.81 to €-33.37).

**Conclusions:**

The POC troponin strategy is likely cost-saving, by reducing hospital referrals. The small increase in missed ACS patients can be partly explained by conservative assumptions used in the analysis. Besides, current developments in POC troponin tests will likely further improve their diagnostic performance. Therefore, future prospective studies are warranted to investigate whether those developments make the POC troponin test to a safe and cost-effective diagnostic tool for diagnosing ACS in general practices.

**Electronic supplementary material:**

The online version of this article (doi:10.1186/s12872-017-0647-6) contains supplementary material, which is available to authorized users.

## Background

Each year almost 200,000 patients in the Netherlands contact their general practitioner (GP) with chest pain [1]. Chest pain is a leading symptom of acute coronary syndrome (ACS), which encompasses both unstable angina pectoris (UAP) and acute myocardial infarction (AMI) [2]. Among AMI patients, two subgroups can be distinguished based on a deviation on the electrocardiogram (ECG), which are ST elevation myocardial infarction (STEMI) and non-ST elevation myocardial infarction (NSTEMI) [2]. However, only approximately 18% of all patients who present with chest pain to the hospital are eventually diagnosed with ACS [3]. Clinical management is a major challenge for GPs, because unnecessary referrals lead to unnecessary costs and patient distress. So, given the potentially life-threatening consequences, accurate and rapid diagnosis of ACS remains crucial.

Besides a patient’s signs, symptoms, medical history, and the information obtained from the ECG, imaging modalities (for example coronary angiography), and laboratory biomarkers of myocardial necrosis (mainly involving high-sensitive troponin), provide additional diagnostic value [2]. As those diagnostic tools are often not readily available in primary care, current clinical guidelines recommend immediate hospital referral when ACS is suspected [4]. However, the introduction of point-of-care (POC) troponin tests, accompanied by a shorter turn-around-time, offers a low-cost diagnostic test for GPs. Although costs per tests are mostly higher for POC tests as compared to regular laboratory tests [5], the use of POC troponin as a diagnostic aid to rule out ACS might improve referral decisions and hence lead to overall cost savings [6]. This advantage needs however to be weighed against the lower sensitivity of this test especially in the early hours after symptom onset [2, 6–8]. Consequently, international guidelines do not give a recommendation on the use of POC troponin [2], while its use is discouraged in Dutch guidelines [9].

In addition to collecting clinical evidence, proper evaluation of the cost-utility of POC troponin testing in primary care is required. Although randomized controlled trials (RCTs) would provide the best source of evidence, these are commonly very expensive, time consuming, and often do not allow assessment of long-term outcomes or risks [10, 11]. Therefore, an early stage health economic model may provide a valid alternative to quantify the effect of POC troponin testing on costs, and on short and long term outcomes. In this paper, we present such model-based health economic analysis of the use of POC troponin testing to diagnose ACS in primary care, as compared to current practice.

## Methods

### Structure of the model

**Fig. 1:**
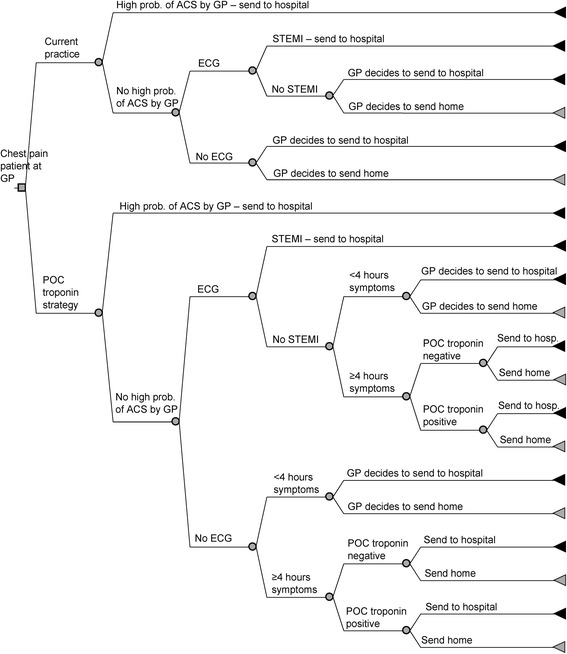
structure of the decision tree. Black triangles represent braches in which patients are referred to the ED (because of suspected ACS), while grey triangles represent branches in which patients are send home. ACS = acute coronary syndrome, ECG = electrocardiogram, GP = general practitioner, hosp. = hospital, POC = point-of-care, ﻿p﻿rob. = proba﻿bility, STEMI = ST elevation myocardial infarction

We developed a patient-level simulation model to reflect the diagnostic pathway of chest pain patients presenting to the GP (Fig. 1). This model was used to analyse the cost-utility of a POC troponin test to exclude ACS in addition to GP examination, as compared to GP examination without the test. As test and treatment decisions in this model are based (partially) on patient’s characteristics and patient’s history (including duration of symptoms, previous test outcomes, and previous management decisions) adequately reflecting the diagnostic pathway of patients requires simulating individual patients. Whereas such decisions are simulated for entire groups in a cohort (state-transition) model, they can be simulated on individual patient level when using a patient-level simulation model. The primary outcome measure was the incremental costs per quality-adjusted life year (QALY) gained for the POC strategy as compared to current practice. Secondary outcome measures were the percentage of chest pain patients that were either correctly or incorrectly referred to the hospital or sent home (expressed as true positives [TPs], true negatives [TNs], false positives [FPs], and false negatives [FNs]). Other secondary outcome measures included mortality and heart failure rates in both strategies. All costs and effects were evaluated from a societal point of view, over a lifelong time horizon.

### Current evidence

The model was populated using data from literature. The following paragraphs present a summary of evidence used, for further details see Additional file 1. An overview of the assumptions used is provided in Additional file 2.

#### Patient pathways

In this patient-level model, each hypothetical patient was assigned an age, gender, and disease state (i.e. ACS or no ACS) based on the population distribution derived from a cross-sectional diagnostic study [12, 13]. For ACS patients in primary care, the probabilities of having either UAP, NSTEMI or STEMI were based on a prospective study [14].

Of all chest pain patients presenting to the GP, 14.7% was immediately referred to the hospital without diagnostic testing, because of a high perceived probability of ACS by the GP (Table 1) [15]. All patients that were referred to the hospital were assumed to be transported by ambulance. Among the patients that were not immediately referred, all GPs who had an ECG in their practice (45%) [16], were assumed to use it in those patients. In case the patient had a STEMI, there was a 50% probability that this was diagnosed in case an ECG was performed [17]. Those patients were also immediately referred to the hospital. In current practice, the GP decides on referral of the remaining patients without POC troponin. The sensitivity and specificity of this decision (i.e. 88.3% and 72.2%, respectively) were derived from Nilsson et al. [18]. For the POC strategy, the time from symptom onset to presentation was estimated for each patient separately [19], and then categorized according to time since symptom onset as a) <4.0 h, b) 4.0–5.5 h, c) 5.5–7.0 h, d) 7.0–10.0 h, and e) >10.0 h. As it is known that the performance of the POC troponin test is less in patients presenting shortly after symptom onset [7], it was assumed that this test was not performed in patients presenting with <4.0 h symptoms [7, 19]. In those patients, the referral decision was based on the judgment of the GP as in current practice [18]. In the remaining patients, it was assumed that the POC troponin test was performed, and the sensitivity and specificity for time categories b), c), d) and e) were derived from Diercks et al. [7]. In case the result of the POC troponin test did not correspond with what would have been the (initial) referral decision of the GP in current practice (i.e. without POC troponin), a probability of the GP revising the initial referral decision was incorporated in the model [15]. In both strategies, all patients who were not immediately referred to the hospital were assumed to be sent home without receiving medical care.

**Table 1:** input parameters concerning patient pathways used in the model

Parameter	Category	Probability	95% CI	Distribution	Reference
Perceived probability of ACS by GP	High probability	14.7%	8.2% to 22.5%	Beta	[15]
ECG	Performed at GP’s office	57.4%	56.0% to 58.7%	Beta	[16]
	Sensitivity for STEMI	50.0%	37.6% to 62.6%	Beta	[17]
Time from symptom onset to presentation (male)	< 4 h	47.5%	38.9% to 55.4%	Dirichlet	[19, 40]
	4–5.5 h	9.0%	5.2% to 14.5%	Dirichlet	[19, 40]
	5.5–7 h	7.3%	3.4% to 11.8%	Dirichlet	[19, 40]
	7–10 h	9.4%	5.2% to 14.9%	Dirichlet	[19, 40]
	> 10 h	26.8%	20.0% to 34.6%	Dirichlet	[19, 40]
Time from symptom onset to presentation (female)	< 4 h	54.8%	47.1% to 62.7%	Dirichlet	[19, 40]
	4–5.5 h	9.4%	5.1% to 13.9%	Dirichlet	[19, 40]
	5.5–7 h	6.5%	3.1% to 10.7%	Dirichlet	[19, 40]
	7–10 h	8.0%	4.6% to 13.1%	Dirichlet	[19, 40]
	> 10 h	21.2%	15.2% to 27.8%	Dirichlet	[19, 40]
Diagnostic performance of GP, without POC troponin	Sensitivity	88.3%	79.2% to 95.2%	Beta	[18]
	Specificity	72.2%	67.9% to 76.3%	Beta	[18]
Sensitivity of POC troponin at different duration of symptoms	4–5.5 h	66.7%	55.5% to 77.0%	Beta	[7]
	5.5–7 h	79.2%	69.3% to 87.5%	Beta	[7]
	7–10 h	84.7%	75.3% to 91.9%	Beta	[7]
	> 10 h	87.5%	78.8% to 94.2%	Beta	[7]
Specificity of POC troponin at different duration of symptoms	4–5.5 h	95.9%	94.2% to 97.3%	Beta	[7]
	5.5–7 h	94.4%	92.5% to 96.1%	Beta	[7]
	7–10 h	93.4%	91.3% to 95.3%	Beta	[7]
	> 10 h	92.6%	90.4% to 94.5%	Beta	[7]
Revise referral decision after discordant POC troponin	Negative troponin	80.6%	73.3% to 87.1%	Beta	[15]
	Positive troponin	83.3%	76.3% to 89.3%	Beta	[15]

This table shows the parameter, the value used in the model, the 95% confidence interval, the distribution used, and the data source. *ACS* acute coronary syndrome, *CI* confidence interval, *ECG* electrocardiogram, *GP* general practitioner, *POC* point-of-care, *STEMI* ST elevation myocardial infarction

Mortality and heart failure rates were dependent on whether patients had ACS, and if present, whether ACS was correctly diagnosed. In addition, as the POC troponin test was assumed to delay time to treatment with 10 min, using this test would increase the mortality rate in AMI patients with 0.2% [20]. This 10 min duration of the POC troponin test was chosen as previous research has shown that GPs consider this the maximum acceptable duration of the POC troponin test [15]. Productivity losses incorporated lost working time due to GP visits, emergency department visits, and due to UAP, STEMI, NSTEMI, heart failure, and mortality [21–23].

#### Quality of life

The quality-adjusted life expectancy (QALE, based on combining expected quality of life with life expectancy) were obtained directly from a systematic review and economic model for STEMI, NSTEMI and UAP patients as well as for healthy patients [24–26]. Separate QALE estimates were used for categories of age and gender [24–26]. A missed diagnosis of ACS indirectly lowered quality of life by increasing the risk of heart failure and mortality. For heart failure patients, the QALEs were estimated by multiplying the reported utility of heart failure patients with their reported average life expectancy [27, 28].

#### Costs

Costs of both a GP consultation and ambulance transportation to the emergency department were derived from the Dutch cost manual [23], whereas costs of performing an ECG were derived from the Dutch Healthcare Authority [29]. As no tariff currently exists for the POC troponin test, and because a troponin test performed in a regular laboratory costs approximately €8 [29], the costs of a POC troponin test were conservatively estimated at €15. Average diagnosis and treatment costs per (suspected) ACS patient were derived from the Dutch Healthcare Authority [3]. Healthcare costs for the remaining lifetime of STEMI, NSTEMI, and UAP patients were derived from Goodacre et al. [24–26]. Lifetime healthcare costs per patient with heart failure were based on Dutch data concerning the point prevalence of heart failure [30], the annual healthcare costs of those patients [31], and combined with the average remaining life expectancy [27]. All costs were converted to 2015 using the consumer price indices as reported by the Dutch Central Bureau of Statistics, and converted to Euros if necessary (exchange rate 1.271 GBP = €1.000, 31 March 2016) [32, 33]. All costs and effects were discounted at an annual rate of 4.0% and 1.5%, respectively, in accordance with Dutch costing guidelines [23]. An overview of all cost parameters, including costs of productivity losses, is shown in Additional file 1.

### Probabilistic sensitivity analysis

A probabilistic sensitivity analysis was performed by means of Monte Carlo simulations, using 10,000 iterations of 20,000 hypothetical, unique patients. Distributions were assigned to all parameters under investigation [34]. An overview of the distributions and the 95% CI for each parameter is provided in Table 1, and Additional file 1.

### Expected value of perfect information (EVPI) analysis

To determine the value of collecting additional information on this topic, and potentially enhance decision-making on the usefulness of POC troponin in primary care a value of information analysis was performed. The EVPI was calculated to estimate the expected costs of uncertainty in the current model, using a value of information tool developed by the University of Sheffield [35].

### Cost-effectiveness acceptability curve (CEAC)

To evaluate the probability that the POC troponin strategy is cost-effective, a CEAC was constructed for a willingness-to-pay (WTP) threshold ranging from €0/QALY to €100,000/QALY. A separate CEAC was constructed under the assumption that use of the POC troponin test is only acceptable when it does not lead to health loss (i.e. does not increase the rate of missed ACS patients) and has acceptable cost-effectiveness.

## Results

### Probabilistic sensitivity analysis

The results indicate that the POC troponin strategy costs on average €1144 (95% CI €892 to €1451) per patient, as compared to €1221 (95% CI €955 to €1541) for current practice. The cost savings for the testing strategy are €77 [95% CI €-127 to €-33] per patient. The probability that patients were correctly not referred to the hospital (TNs) would increase from 57.90% to 64.51% (+6.61%). Consequently, unnecessary referrals (FPs) decreased with 6.61% (i.e. from 38.46% to 31.85%). However, the probability of patients being correctly referred to the hospital (TPs) showed a very small decrease from 3.42% to 3.37% (i.e. -0.05%), which in turn resulted in an increase of patients incorrectly not referred (FNs) from 0.22% to 0.27% (i.e. 54 vs. 45 per 20,000 simulated patients). In turn, this results in a very small increase in the probability of patients dying of ACS (i.e. 61 vs. 60 per 20,000 patients), as well as a small increase in the incidence of heart failure (i.e. 121 vs. 119 per 20,000 patients). The overall change in QALYs from POC troponin use is negligible (−0.0004 when rounded to 4 decimals, i.e. 3.5 h in full health).

**Fig. 2:**
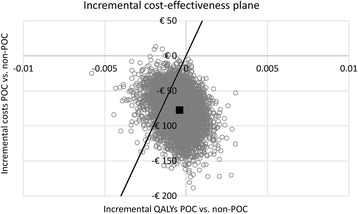
Incremental cost-effectiveness plane, showing the result of 10,000 model iterations of 20,000 patients, the mean value, and the ICUR threshold of €20,000/QALY. ICUR = incremental cost-utility r﻿atio﻿﻿, POC = point-of-care, QALY = quality-adjusted life year

Given the negligible impact on health outcomes, it is not useful to calculate an incremental cost utility ratio (ICUR), as this outcome is very unstable for tiny differences in health outcomes as estimated in this study. When combining differences in health outcomes with differences in costs, an incremental net monetary benefit of €69 was found. In the POC strategy, the POC troponin test was used in approximately 40% of all chest pain patients presenting to their GP. This strategy could result in annual cost savings in the Netherlands of approximately €14 million [1]. A detailed overview of all model outcomes, including the 95% CI, is shown in Table 2, and the incremental cost-effectiveness plane is shown in Fig. 2. In Table 2, the costs are divided in four categories: primary care costs involves the costs from presentation at the GP including potential hospital referral by ambulance. Hospital costs involves the costs from hospital admission until discharge. Lifetime costs involves the costs of follow-up after STEMI. Also, the effect on (correct) referral decisions, as well as the effect on the probability of heart failure and mortality is shown*.*

**Table 2:** Outcomes of Monte Carlo simulations

Parameter	Without POC troponin (95% CI)	With POC troponin (95% CI)	Absolute effect (POC vs. non-POC) (95% CI)	Relative effect (POC vs. non-POC) (95% CI)
Primary care costs per patient	€290.52 (€188.99 to €421.09)	€262.10 (€173.63 to €375.33)	€-28.41 (€-50.38 to €-12.23)	−9.78% (−13.36% to −5.75%)
Hospital costs per patient	€446.00 (€307.80 to €620.41)	€398.38 (€277.19 to €551.81)	€-47.62 (€-77.28 to €-25.85)	−10.68% (−14.27% to −7.12%)
Lifetime costs per patient	€377.55 (€242.56 to €554.17)	€384.13 (€245.85 to €565.96)	€6.58 (€-17.25 to €33.47)	1.74% (−4.49% to 8.86%)
Costs of productivity loss per patient	€106.87 (€64.71 to €169.27)	€99.07 (€59.52 to €160.09)	€-7.80 (€-18.76 to €2.58)	−7.30% (−16.94% to 2.70%)
Total costs per patient	€1220.94 (€955.23 to €1540.85)	€1143.68 (€891.74 to €1451.12)	€ -77.25 (€-126.81 to €-33.37)	−6.33% (−9.80% to −2.85%)
Quality adjusted life expectancy	11.29 (9.41 to 13.33)	11.29 (9.41 to 13.33)	﻿−0.00 (−0.00 to 0.00)	−0.00% (−0.02% to 0.01%)
Probability TP	3.42% (2.47% to 4.53%)	3.37% (2.44% to 4.47%)	−0.05% (−0.15% to 0.06%)	−1.37% (−4.08% to 1.64%)
Probability FP	38.46% (33.97% to 45.00%)	31.85% (28.29% to 38.70%)	−6.61% (−8.30% to −4.99%)	−17.19% (−20.86% to −12.66%)
Probability TN	57.90% (51.52% to 62.38%)	64.51% (57.82% to 68.14%)	6.61% (4.99% to 8.30%)	11.42% (8.34% to 15.06%)
Probability FN	0.22% (0.08% to 0.46%)	0.27% (0.14% to 0.46%)	0.05% (−0.06% to 0.15%)	20.91% (−15.72% to 135.02%)
Probability death	0.30% (0.19% to 0.45%)	0.30% (0.19% to 0.46%)	0.00% (−0.01% to 0.03%)	1.53% (−4.11% to 9.30%)
Probability HF	0.59% (0.40% to 0.83%)	0.60% (0.41% to 0.84%)	0.01% (−0.03% to 0.05%)	1.85% (−4.24% to 8.91%)

This table shows the results of 10,000 iterations of 20,000 patients, showing the costs and remaining quality-adjusted life expectancy per patient. Both the 95% CI and the absolute and relative effect are provided. *CI* confidence interval, *FN* false negative, *FP* false positive, *HF* heart failure, *POC* point-of-care, *TN* true negative, *TP* true positive

More specifically, the average cost savings of €77.25 per patient are mainly attributable to a decreased referral rate, resulting in a reduction in costs in primary care with €28.41 per patient (as caused by a decrease in hospital referrals by ambulance), accompanied by a decrease in costs of hospital treatment of €47.62 per patient. Although the lifetime costs per patient are slightly higher (i.e. +€6.58), which is mainly attributable to a small increase in heart failure rates, those higher costs are compensated by a decrease in productivity loss per patient (−€7.80), caused by a decrease in hospital referral rates.

### EVPI results

Results indicate that the overall EVPI per person presenting with chest pain in primary care is estimated to be €0.22. Based on a decision relevance of ten years, and an estimated 182,897 individuals presenting with chest pain in this period, this results in an overall EVPI per year of €403,364 in the Netherlands [1]. Thus, giving this low EVPI, it is unlikely that performing more research, to decrease uncertainty, would be considered an efficient use of resources. An overview of the expected value of partial perfect information for groups of model input parameters is shown in Additional file 3.

### CEAC results

**Fig. 3:**
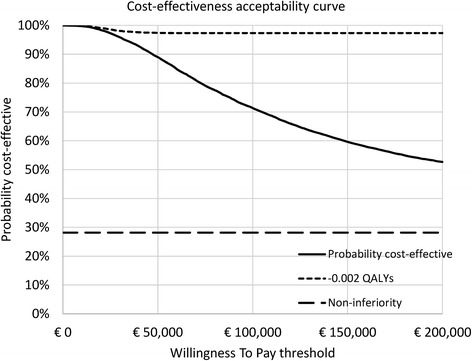
Cost-effectiveness acceptability curve of the POC troponin strategy versus current practice, for a WTP ranging from €0/QALY to €200,000/QALY. The black line represents the base case cost-effectiveness acceptability curve. The ‘non-inferiority’ and ‘-0.002 QALYs’ line represent scenarios in which POC troponin has acceptable cost-utility, and is either at least equally effective, or does not involve a decrease of ≥0.002 QALYs. QALYs = quality-adjusted life years

The CEAC indicates that the probability of the POC troponin strategy to be cost-effective ranges from 100.0% to 52.7%, for a WTP threshold of €0 to €200,000/QALY. For a WTP of €20,000/QALY, this probability was 98.4% (Fig. 3). Thus, when society is unwilling to pay any money for an increase in health outcomes (i.e. a WTP of €0/QALY), the POC troponin strategy has to be cheaper compared to current practice to be considered cost-effective, regardless of the effect on health outcomes. On the other hand, a very high WTP indicates that society is unwilling to accept any loss in QALYs. Therefore, an increase in WTP decreases the probability that the POC troponin strategy is cost-effective. However, when the POC test was also required to provide equal or better health outcomes compared with usual care (non-inferiority), the probability of the POC troponin strategy to be cost-effective remained stable at 28.2% for a WTP ranging from €0 to €200,000/QALY (Fig. 3). Although this suggests that the POC troponin strategy has a large chance of resulting in health loss, it should be noted that the actual absolute health loss is likely to be vanishingly small. For example, previous research has indicated that a change in health utility of 0.03 is the minimum clinically important difference, and that smaller differences can be considered to be not clinically important [36]. In our analysis the difference in utility is much smaller than 0.03, as even over the lifetime horizon the accumulated corresponding health loss equals only −0.0004. Indeed, when a very small decrease of 0.002 QALYs is chosen as cut-off for the maximum acceptable health loss, the probability that the POC troponin strategy is cost-effective already remains very high, decreasing only from 100.0% to 97.3% for a WTP ranging from €0 to €200,000/QALY (Fig. 3).

## Discussion

Using a POC troponin test in primary care is expected to increase the probability at which the GP is able to correctly exclude ACS (TNs), accompanied by a decrease in unnecessary hospital referrals (FPs). However, the rate of correct referrals (TPs) decreases slightly, while the rate at which ACS patients are incorrectly not referred (FNs) shows a small increase. Despite this, the average per patient effect on QALYs is very small (i.e. -0.0004). Costs per chest pain patient are expected to decrease with approximately €77, indicating that the POC troponin strategy is likely cost-saving as compared to current practice.

### Strengths

The main strength of the current study is that it was designed to accurately represent the current work-up of GPs, in chest pain patients. To achieve this, three real decisions were built into the model, in which the POC troponin test is unlikely to be used: 1) when the GPs considers a patient at high risk of ACS, 2) when the GP diagnoses STEMI using an ECG, or 3) when a patient presents <4 h after symptom onset. Consequently, the POC troponin test is only used in approximately 40% of all chest pain patients presenting to the GP. Furthermore, as it is unlikely that GPs base their referral decision solely on a POC troponin test result, the probability of GPs adhering to this result was also incorporated. Although those assumptions inevitably limit the potential impact of the POC troponin test, they are crucial to obtain a realistic estimate of the test’s potential cost-utility.

Secondly, the use of a patient-level model has several advantages. First of all, probabilities of ACS are both age and gender specific, allowing to estimate costs, QALYs and productivity losses at the individual patient level. In addition, a duration of symptoms was assigned to each patient, allowing to incorporate time-dependent sensitivities and specificities, which is an important characteristic of the POC troponin test [7].

Furthermore, all assumptions used in the analysis were conservative regarding the potential impact of POC troponin, which may have led to underestimating both cost savings and health benefits. First, the delay by POC troponin testing was set at 10 min, thereby assuming that the test is performed after the consultation by the GP, whereas it is likely that the test can already be performed during the patient’s anamnesis and physical examination. Besides cost savings, this work-up would also prevent the potential negative health impact caused by a delay in treatment. Second, it was assumed that GPs would perform the test in all remaining chest pain patients, and therefore also in patients that are not actually suspected of having ACS. However, GPs can probably exclude ACS with certainty in some patients without using the POC troponin test, thereby saving costs. Third, it was assumed that the 14.7% of patients that are perceived to be at high risk of ACS by their GP (and who are immediately referred), corresponded to the patients with the highest actual probability of ACS. This assumption most likely overestimates the ability of the GP to identify high-risk patients.

### Limitations

There are two limitations to be mentioned. First, evidence on the correlation between several input parameters were lacking. For example, no evidence was available on the relationship between a) the pre-test probability of ACS, b) diagnostic accuracy of the GP in current practice, and c) the diagnostic accuracy of the POC troponin test. Similarly, no evidence was available on the potential influence of patient’s characteristics, such as age and gender, on the probability that the GP revises his or her initial referral decision following a discordant POC troponin result. Therefore, changes in referral decisions made in practice may concern different patients than those with a change in referral decision in our model. However, results of a systematic review and meta-analysis indicate that it was not possible to define an important role for signs and symptoms in diagnosing AMI or ACS [37]. Accordingly, it is unlikely that this limitation strongly affects model outcomes.

Second, as this study is based on a hypothetical cohort of patients, the impact of POC troponin testing on health outcomes is dependent on the literature used in the model. However, evidence regarding the diagnostic performance of GPs (without the use of POC) is limited. The drawback of the study used in the current analysis, is that the sensitivity of the GP was based on their ‘action in daily practice’ in chest pain patients. This definition however, included besides referral to the hospital, also exercise testing, prescribing medication, and requesting a second opinion [18]. In addition, the diagnostic performance may also be dependent on the type of POC analyzer used, but the number of studies that report the diagnostic performance of POC troponin for (a) specific time(s) since symptom onset is limited. Furthermore, more sensitive POC troponin tests have already become available [38]. All in all, the current model may thus have overestimated the rate of missed ACS patients due to POC troponin use. In absence of real-life patient data, this model-based analysis gives a first insight in to the potential cost-effectiveness of the POC troponin test. Our results indicate that future prospective studies into the safety and efficacy of using POC troponin in primary care are warranted.

### Implications for practice

Results from a previous study indicate that GPs’ main requirements regarding POC troponin tests are a) test result within 10 min, b) a test performed with a finger prick, and c) reimbursement of the POC analyzer [15]. Recently developed POC troponin tests may already comply with the desired turn-around-time and the finger prick blood sample [39]. In addition, literature indicates that the diagnostic performance of recently developed POC troponin tests is comparable to the results obtained in a routine laboratory. Consequently, the accompanying increase in negative predictive value likely prevents that ACS patients are missed, thereby making use of the POC troponin test a cost-effective strategy. In turn, further research is necessary to investigate whether this lower limit of detection may allow use of this test to rule out ACS in patients presenting within four hours after symptom onset. In addition, including the expected improvement on patient’s well-being (and consequently on QALYs) as achieved by decreasing unnecessary hospital referrals would further increase the potential benefit of this test.

## Conclusion

Owing to the expected reduction in hospital referrals, the POC troponin strategy is likely cost-saving. The potential (accompanying) small increase in missed ACS patients can partly be explained by conservative assumptions used in the analysis. In addition, ongoing developments in POC troponin tests will likely further improve their diagnostic performance. Therefore, the use of a POC troponin testing strategy is likely to become cost-effective in the nearby future. When data from new prospective (randomized) studies into the safety and diagnostic accuracy of the use of POC troponin tests become available, the health economic model presented here allows rapid reassessment into the benefits of such a diagnostic tool for diagnosing ACS in general practices.

## Additional files

Additional file 1: Model input parameters. This file contains an overview of all model input parameters, including the value used in the model, the 95% confidence interval, the distribution used, and the data source (DOCX 73 kb)

Additional file 2: Overview of model assumptions. This file contains an overview of all assumptions that have been used in the health economic analysis (DOCX 18 kb)

Additional file 3: Results of partial perfect value of information analysis. This file contains an overview of the results of the partial perfect value of information analysis (DOCX 13 kb)

## Abbreviations

ACS: acute coronary syndrome
CEAC: cost-effectiveness acceptability curve
CI: confidence interval
ECG: electrocardiogram
EVPI: expected value of perfect information
FN: false negative
FP: false positive
GP: general practitioner
NSTEMI: non-ST elevation myocardial infarction
POC: point-of-care
QALY: quality-adjusted life year
RCT: randomized controlled trial
STEMI: ST elevation myocardial infarction
TN: true negative
TP: true positive
UAP: unstable angina pectoris
WTP: willingness-to-pay
