# Supplementary material for: The cost-utility of point-of-care troponin testing to diagnose acute coronary syndrome in primary care
Source: BMC Cardiovasc Disord. 2017 Aug 2;17:213. doi: 10.1186/s12872-017-0647-6 (PMC5541723; doi:10.1186/s12872-017-0647-6)
Supplement: Supplementary file 1 — Model input parameters. This file contains an overview of all model input parameters, including the value used in the model, the 95% confidence interval, the distribution used, and the data source (DOCX 73 kb) [file 12872_2017_647_MOESM1_ESM.docx]

**Additional file 1: model input parameters**

**Table 1a.** model input parameters concerning patient pathways used in the model, showing the parameter, the value used in the model, the 95% confidence interval, the distribution used, and the data source. ACS = acute coronary syndrome, AMI = acute myocardial infarction, CI = confidence interval, ECG = electrocardiogram, ED = emergency department, GP = general practitioner, HF = heart failure, NSTEMI = non-ST elevation myocardial infarction, POCT = point-of-care test, STEMI = ST elevation myocardial infarction, UAP = unstable angina pectoris.

*Calculated probabilities based on the total occurrence of ACS, and the age and gender specific probabilities of patients presenting to the GP with chest pain.

| **Parameter** | **Category** | **Probability** | **95% CI** | **Distribution** | **Reference** |
| --- | --- | --- | --- | --- | --- |
| Population age distribution of chest pain patients at the GP (male) | 35 – 44 years | 21.2% | 17.9% to 24.9% | Dirichlet | ([1](#_ENREF_1)) |
|  | 45 – 54 years | 21.3% | 17.7% to 24.5% | Dirichlet | ([1](#_ENREF_1)) |
|  | 55 – 64 years | 20.8% | 17.6% to 24.5% | Dirichlet | ([1](#_ENREF_1)) |
|  | 65 – 74 years | 23.4% | 19.9% to 27.0% | Dirichlet | ([1](#_ENREF_1)) |
|  | ≥ 75 years | 13.3% | 10.6% to 16.3% | Dirichlet | ([1](#_ENREF_1)) |
| Population age distribution of chest pain patients at the GP (female) | 35 – 44 years | 17.0% | 14.3% to 19.9% | Dirichlet | ([1](#_ENREF_1)) |
|  | 45 – 54 years | 20.5% | 17.4% to 23.5% | Dirichlet | ([1](#_ENREF_1)) |
|  | 55 – 64 years | 18.3% | 15.4% to 21.3% | Dirichlet | ([1](#_ENREF_1)) |
|  | 65 – 74 years | 25.4% | 22.4% to 29.0% | Dirichlet | ([1](#_ENREF_1)) |
|  | ≥ 75 years | 18.9% | 16.0% to 21.8% | Dirichlet | ([1](#_ENREF_1)) |
| Probability of ACS for age (male) | 35 – 44 years | 1.7% | 0.2% to 4.6% | Beta | ([2](#_ENREF_2))* |
|  | 45 – 54 years | 1.5% | 0.1% to 4.5% | Beta | ([2](#_ENREF_2))* |
|  | 55 – 64 years | 3.8% | 1.1% to 8.1% | Beta | ([2](#_ENREF_2))* |
|  | 65 – 74 years | 7.1% | 3.3% to 12.1% | Beta | ([2](#_ENREF_2))* |
|  | ≥ 75 years | 10.3% | 4.4% to 18.4% | Beta | ([2](#_ENREF_2))* |
| Probability of ACS for age (female) | 35 – 44 years | 1.0% | 0.0% to 3.4% | Beta | ([2](#_ENREF_2))* |
|  | 45 – 54 years | 0.9% | 0.0% to 2.9% | Beta | ([2](#_ENREF_2))* |
|  | 55 – 64 years | 2.2% | 0.4% to 5.4% | Beta | ([2](#_ENREF_2))* |
|  | 65 – 74 years | 4.2% | 1.8% to 7.6% | Beta | ([2](#_ENREF_2))* |
|  | ≥ 75 years | 6.0% | 2.6% to 10.7% | Beta | ([2](#_ENREF_2))* |
| Suspected ACS gender distribution | Male | 43.9% | 41.3% to 46.8% | Beta | ([1](#_ENREF_1)) |
| Time from symptom onset to presentation (male) | < 4 hours | 47.5% | 38.9% to 55.4% | Dirichlet | ([3](#_ENREF_3), [4](#_ENREF_4)) |
|  | 4 – 5.5 hours | 9.0% | 5.2% to 14.5% | Dirichlet | ([3](#_ENREF_3), [4](#_ENREF_4)) |
|  | 5.5 – 7 hours | 7.3% | 3.4% to 11.8% | Dirichlet | ([3](#_ENREF_3), [4](#_ENREF_4)) |
|  | 7 – 10 hours | 9.4% | 5.2% to 14.9% | Dirichlet | ([3](#_ENREF_3), [4](#_ENREF_4)) |
|  | > 10 hours | 26.8% | 20.0% to 34.6% | Dirichlet | ([3](#_ENREF_3), [4](#_ENREF_4)) |
| Time from symptom onset to presentation (female) | < 4 hours | 54.8% | 47.1% to 62.7% | Dirichlet | ([3](#_ENREF_3), [4](#_ENREF_4)) |
|  | 4 – 5.5 hours | 9.4% | 5.1% to 13.9% | Dirichlet | ([3](#_ENREF_3), [4](#_ENREF_4)) |
|  | 5.5 – 7 hours | 6.5% | 3.1% to 10.7% | Dirichlet | ([3](#_ENREF_3), [4](#_ENREF_4)) |
|  | 7 – 10 hours | 8.0% | 4.6% to 13.1% | Dirichlet | ([3](#_ENREF_3), [4](#_ENREF_4)) |
|  | > 10 hours | 21.2% | 15.2% to 27.8% | Dirichlet | ([3](#_ENREF_3), [4](#_ENREF_4)) |
| ECG | Performed at GP’s office | 57.4% | 56.0% to 58.7% | Beta | ([5](#_ENREF_5)) |
|  | Sensitivity for STEMI | 50.0% | 37.6% to 62.6% | Beta | ([6](#_ENREF_6)) |
| Perceived probability of ACS by GP | High probability | 14.7% | 8.2% to 22.5% | Beta | (submitted, [7](#_ENREF_7)) |
| Revise referral decision after discordant POCT troponin | Negative troponin | 80.6% | 73.3% to 87.1% | Beta | (submitted, [7](#_ENREF_7)) |
|  | Positive troponin | 83.3% | 76.3% to 89.3% | Beta | (submitted, [7](#_ENREF_7)) |
| Diagnostic performance of GP, without POCT troponin | Sensitivity | 88.3% | 79.2% to 95.2% | Beta | ([8](#_ENREF_8)) |
|  | Specificity | 72.2% | 67.9% to 76.3% | Beta | ([8](#_ENREF_8)) |
| Sensitivity of POC troponin at different duration of symptoms | 4 – 5.5 hours | 66.7% | 55.5% to 77.0% | Beta | ([9](#_ENREF_9)) |
|  | 5.5 – 7 hours | 79.2% | 69.3% to 87.5% | Beta | ([9](#_ENREF_9)) |
|  | 7 – 10 hours | 84.7% | 75.3% to 91.9% | Beta | ([9](#_ENREF_9)) |
|  | > 10 hours | 87.5% | 78.8% to 94.2% | Beta | ([9](#_ENREF_9)) |
| Specificity of POC troponin at different duration of symptoms | 4 – 5.5 hours | 95.9% | 94.2% to 97.3% | Beta | ([9](#_ENREF_9)) |
|  | 5.5 – 7 hours | 94.4% | 92.5% to 96.1% | Beta | ([9](#_ENREF_9)) |
|  | 7 – 10 hours | 93.4% | 91.3% to 95.3% | Beta | ([9](#_ENREF_9)) |
|  | > 10 hours | 92.6% | 90.4% to 94.5% | Beta | ([9](#_ENREF_9)) |
| Mortality modifiers | Per 10 years increase in age | 1.7 | 1.6 to 1.9 | LogNormal | ([10](#_ENREF_10)) |
|  | Missed UAP | 1.7 | 0.2 to 15.2 | LogNormal | ([11](#_ENREF_11)) |
|  | Missed NSTEMI | 1.9 | 0.7 to 5.2 | LogNormal | ([11](#_ENREF_11)) |
|  | Missed STEMI | 1.9 | 0.7 to 5.2 | LogNormal | ([11](#_ENREF_11)) |
|  | Per 10 minutes delay due to POCT troponin – NSTEMI | 0.2% | 0.1% to 0.4% | Beta | ([12](#_ENREF_12)) |
|  | Per 10 minutes delay due to POCT troponin – STEMI | 0.2% | 0.1% to 0.4% | Beta | ([12](#_ENREF_12)) |
| Occurrence of each type of ACS (among ACS) | UAP or NSTEMI | 72.7% | 61.5% to 82.5% | Beta | ([13](#_ENREF_13)) |
|  | UAP (among UAP or NSTEMI) | 29.2% | 17.3% to 42.5% | Beta | ([13](#_ENREF_13)) |
| Death at ED | Diagnosed UAP | 2.7% | 2.4% to 3.1% | Beta | ([14](#_ENREF_14)) |
|  | Diagnosed NSTEMI | 5.9% | 5.4% to 6.4% | Beta | ([14](#_ENREF_14)) |
|  | Diagnosed STEMI | 7.8% | 7.2% to 8.4% | Beta | ([14](#_ENREF_14)) |
| Heart failure | Diagnosed UAP | 9.2% | 8.5% to 10.0% | Beta | ([14](#_ENREF_14)) |
|  | Missed UAP | 44.4% | 23.0% to 67.0% | Beta | ([15](#_ENREF_15)) |
|  | Diagnosed NSTEMI | 17.9% | 16.9% to 18.9% | Beta | ([14](#_ENREF_14)) |
|  | Missed NSTEMI | 44.4% | 23.0% to 66.4% | Beta | ([15](#_ENREF_15)) |
|  | Diagnosed STEMI | 18.4% | 17.4% to 19.4% | Beta | ([14](#_ENREF_14)) |
|  | Missed STEMI | 44.4% | 22.7% to 66.7% | Beta | ([15](#_ENREF_15)) |
| Production hours lost due to UAP, NSTEMI, STEMI, HF or death | UAP | 91.4 | 51.6 to 142.1 | Gamma | ([16](#_ENREF_16)) |
|  | NSTEMI | 71.7 | 41.0 to 111.3 | Gamma | ([16](#_ENREF_16)) |
|  | STEMI | 71.7 | 41.0 to 111.3 | Gamma | ([16](#_ENREF_16)) |
|  | UAP/NSTEMI/STEMI + HF | 408.9 | 233.6 to 638.1 | Gamma | ([17](#_ENREF_17)) |
|  | Death | 675.1 | 389.1 to 1045.9 | Gamma | ([18](#_ENREF_18)) |
| Working hours lost due to GP and possibly ED visit (non ACS patients) | GP, referral | 8.0 | 4.6 to 12.5 | Gamma | Assumption, ([18](#_ENREF_18)) |
|  | GP, no referral, no POCT | 0.8 | 0.5 to 1.3 | Gamma | Assumption, ([18](#_ENREF_18)) |
|  | GP, no referral, POCT | 0.7 | 0.4 to 1.0 | Gamma | Assumption, ([18](#_ENREF_18)) |

**Table 1b**. Quality of life estimates used in the model, showing the parameter, the value used in the model, the 95% confidence interval, the distribution used, and the data source. ACS = acute coronary syndrome, AMI = acute myocardial infarction, CI = confidence interval, HF = heart failure, NSTEMI = non-ST elevation myocardial infarction, STEMI = ST elevation myocardial infarction, UAP = unstable angina pectoris, QALY = quality-adjusted life year.

* 95% CI is based on an assumed standard error of 25%.

| **Parameter** | **Category** | **QALYs** | **95% CI *** | **Distribution** | **Reference** |
| --- | --- | --- | --- | --- | --- |
| Lifetime QALYs AMI (NSTEMI or STEMI) | 35 – 44 years | 12.2 | 6.2 to 18.2 | Gamma | ([19](#_ENREF_19)) |
|  | 45 – 54 years | 9.5 | 4.8 to 14.1 | Gamma | ([19](#_ENREF_19)) |
|  | 55 – 64 years | 6.7 | 3.4 to 10.0 | Gamma | ([19](#_ENREF_19)) |
|  | 65 – 74 years | 4.7 | 2.4 to 6.9 | Gamma | ([19](#_ENREF_19)) |
|  | ≥ 75 years | 2.4 | 1.2 to 3.6 | Gamma | ([19](#_ENREF_19)) |
| Lifetime QALYs UAP | 35 – 44 years | 13.0 | 6.6 to 19.4 | Gamma | ([19-21](#_ENREF_19)) |
|  | 45 – 54 years | 10.2 | 5.2 to 15.2 | Gamma | ([19-21](#_ENREF_19)) |
|  | 55 – 64 years | 7.3 | 3.7 to 10.9 | Gamma | ([19-21](#_ENREF_19)) |
|  | 65 – 74 years | 5.4 | 2.7 to 8.0 | Gamma | ([19-21](#_ENREF_19)) |
|  | ≥ 75 years | 3.1 | 1.6 to 4.6 | Gamma | ([19-21](#_ENREF_19)) |
| Lifetime QALYs without ACS (males) | 35 – 44 years | 19.5 | 9.9 to 29.0 | Gamma | ([19](#_ENREF_19)) |
|  | 45 – 54 years | 15.8 | 8.1 to 23.6 | Gamma | ([19](#_ENREF_19)) |
|  | 55 – 64 years | 11.5 | 5.9 to 17.1 | Gamma | ([19](#_ENREF_19)) |
|  | 65 – 74 years | 7.6 | 3.9 to 11.3 | Gamma | ([19](#_ENREF_19)) |
|  | ≥ 75 years | 3.3 | 1.7 to 4.9 | Gamma | ([19](#_ENREF_19)) |
| Lifetime QALYs without ACS (females) | 35 – 44 years | 19.0 | 9.7 to 28.3 | Gamma | ([19](#_ENREF_19)) |
|  | 45 – 54 years | 15.4 | 7.9 to 23.0 | Gamma | ([19](#_ENREF_19)) |
|  | 55 – 64 years | 11.2 | 5.7 to 16.7 | Gamma | ([19](#_ENREF_19)) |
|  | 65 – 74 years | 7.4 | 3.8 to 11.0 | Gamma | ([19](#_ENREF_19)) |
|  | ≥ 75 years | 3.2 | 1.6 to 4.7 | Gamma | ([19](#_ENREF_19)) |
| Lifetime QALYs HF (males) | 35 – 44 years | 9.9 | 5.1 to 14.8 | Gamma | ([22](#_ENREF_22), [23](#_ENREF_23)) |
|  | 45 – 54 years | 7.5 | 3.8 to 11.2 | Gamma | ([22](#_ENREF_22), [23](#_ENREF_23)) |
|  | 55 – 64 years | 5.0 | 2.6 to 7.5 | Gamma | ([22](#_ENREF_22), [23](#_ENREF_23)) |
|  | 65 – 74 years | 3.4 | 1.8 to 5.1 | Gamma | ([22](#_ENREF_22), [23](#_ENREF_23)) |
|  | ≥ 75 years | 2.5 | 1.3 to 3.8 | Gamma | ([22](#_ENREF_22), [23](#_ENREF_23)) |
| Lifetime QALYs HF (females) | 35 – 44 years | 9.8 | 5.0 to 14.6 | Gamma | ([22](#_ENREF_22), [23](#_ENREF_23)) |
|  | 45 – 54 years | 7.5 | 3.8 to 11.1 | Gamma | ([22](#_ENREF_22), [23](#_ENREF_23)) |
|  | 55 – 64 years | 5.1 | 2.6 to 7.5 | Gamma | ([22](#_ENREF_22), [23](#_ENREF_23)) |
|  | 65 – 74 years | 3.4 | 1.8 to 5.1 | Gamma | ([22](#_ENREF_22), [23](#_ENREF_23)) |
|  | ≥ 75 years | 2.5 | 1.3 to 3.7 | Gamma | ([22](#_ENREF_22), [23](#_ENREF_23)) |

**Table 1c.** Cost parameters used in the model, showing the parameter, the value used in the model, the 95% confidence interval, the distribution used, and the data source. ACS = acute coronary syndrome, AMI = acute myocardial infarction, CI = confidence interval, ECG = electrocardiogram, ED = emergency department, GP = general practitioner, HF = heart failure, NSTEMI = non-ST elevation myocardial infarction, POCT = point-of-care test, STEMI = ST elevation myocardial infarction, UAP = unstable angina pectoris. All costs >€1,000 are rounded to zero decimals.

* 95% CI is based on an assumed standard error of 25%.

| **Parameter** | **Category** | **Costs** | **95% CI*** | **Distribution** | **Reference** |
| --- | --- | --- | --- | --- | --- |
| Costs at GP office and ambulance transport | GP consultation (10 minutes) | €33.21 | €18.84 to €51.36 | Gamma | ([18](#_ENREF_18)) |
|  | ECG at GP office | €9.59 | €5.52 to €14.91 | Gamma | ([24](#_ENREF_24)) |
|  | POCT troponin at GP | €15.00 | €8.66 to €23.38 | Gamma | Assumption |
|  | Ambulance to ED | €518.33 | €295.93 to €802.29 | Gamma | ([18](#_ENREF_18)) |
| Diagnostic and treatment costs | UAP treatment | €3,042 | €1,782 to €4,687 | Gamma | ([25](#_ENREF_25)) |
|  | NSTEMI treatment | €4,878 | €2,812 to €7,479 | Gamma | ([25](#_ENREF_25)) |
|  | STEMI treatment | €5,771 | €3,355 to €8,893 | Gamma | ([25](#_ENREF_25)) |
|  | Diagnostics ED (no ACS) | €717.57 | €414.89 to €1,103 | Gamma | ([25](#_ENREF_25)) |
| Discounted lifetime costs AMI patients | 30 – 44 years | €5,185 | €2,943 to €7,992 | Gamma | ([19](#_ENREF_19)) |
|  | 45 – 54 years | €4,025 | €2,276 to €6,248 | Gamma | ([19](#_ENREF_19)) |
|  | 55 – 64 years | €2,862 | €1,650 to €4,416 | Gamma | ([19](#_ENREF_19)) |
|  | 65 – 74 years | €1,977 | €1,145 to €3,061 | Gamma | ([19](#_ENREF_19)) |
|  | ≥ 75 years | €1,034 | €597.70 to €1,603 | Gamma | ([19](#_ENREF_19)) |
| Discounted lifetime costs UAP patients | 30 – 44 years | €5,453 | €3,115 to €8,391 | Gamma | ([19-21](#_ENREF_19)) |
|  | 45 – 54 years | €4,271 | €2,420 to €6,606 | Gamma | ([19-21](#_ENREF_19)) |
|  | 55 – 64 years | €3,059 | €1,776 to €4,709 | Gamma | ([19-21](#_ENREF_19)) |
|  | 65 – 74 years | €2,261 | €1,296 to €3,482 | Gamma | ([19-21](#_ENREF_19)) |
|  | ≥ 75 years | €1,295 | €736.99 to €2,018 | Gamma | ([19-21](#_ENREF_19)) |
| Discounted lifetime costs HF patients (male) | 30 – 44 years | €113,073 | €63,447 to €176,649 | Gamma | ([23](#_ENREF_23), [26](#_ENREF_26), [27](#_ENREF_27)) |
|  | 45 – 54 years | €89,827 | €51,944 to €139,300 | Gamma | ([23](#_ENREF_23), [26](#_ENREF_26), [27](#_ENREF_27)) |
|  | 55 – 64 years | €63,619 | €35,998 to €98,820 | Gamma | ([23](#_ENREF_23), [26](#_ENREF_26), [27](#_ENREF_27)) |
|  | 65 – 74 years | €44,757 | €25,580 to €68,797 | Gamma | ([23](#_ENREF_23), [26](#_ENREF_26), [27](#_ENREF_27)) |
|  | ≥ 75 years | €33,481 | €18,957 to €51,527 | Gamma | ([23](#_ENREF_23), [26](#_ENREF_26), [27](#_ENREF_27)) |
| Discounted lifetime costs HF patients (female) | 30 – 44 years | €112,354 | €64,477 to €171,333 | Gamma | ([23](#_ENREF_23), [26](#_ENREF_26), [27](#_ENREF_27)) |
|  | 45 – 54 years | €89,624 | €50,804 to €138,934 | Gamma | ([23](#_ENREF_23), [26](#_ENREF_26), [27](#_ENREF_27)) |
|  | 55 – 64 years | €63,735 | €36,442 to €99,045 | Gamma | ([23](#_ENREF_23), [26](#_ENREF_26), [27](#_ENREF_27)) |
|  | 65 – 74 years | €44,686 | €25,219 to €69,384 | Gamma | ([23](#_ENREF_23), [26](#_ENREF_26), [27](#_ENREF_27)) |
|  | ≥ 75 years | €33,083 | €18,879 to €51,394 | Gamma | ([23](#_ENREF_23), [26](#_ENREF_26), [27](#_ENREF_27)) |
| Costs of production loss per hour (male) | 35 – 45 years | €33.41 | €19.18 to €51.13 | Gamma | ([18](#_ENREF_18)) |
|  | 45 – 55 years | €36.56 | €20.89 to €56.94 | Gamma | ([18](#_ENREF_18)) |
|  | 55 – 65 years | €37.10 | €20.81 to €57.74 | Gamma | ([18](#_ENREF_18)) |
| Costs of production loss per hour (female) | 35 – 45 years | €27.55 | €15.71 to €42.47 | Gamma | ([18](#_ENREF_18)) |
|  | 45 – 55 years | €27.48 | €15.97 to €42.56 | Gamma | ([18](#_ENREF_18)) |
|  | 55 – 65 years | €27.48 | €15.68 to €42.12 | Gamma | ([18](#_ENREF_18)) |

**References**

1. Bosner S, Haasenritter J, Hani MA, Keller H, Sonnichsen AC, Karatolios K, et al. Gender differences in presentation and diagnosis of chest pain in primary care. BMC Fam Pract 2009;10:79.

2. Bosner S, Becker A, Haasenritter J, Abu Hani M, Keller H, Sonnichsen AC, et al. Chest pain in primary care: Epidemiology and pre-work-up probabilities. Eur J Gen Pract 2009;15:141-6.

3. Bruins Slot MH, Rutten FH, van der Heijden GJ, Doevendans PA, Mast EG, Bredero AC, et al. Gender differences in pre-hospital time delay and symptom presentation in patients suspected of acute coronary syndrome in primary care. Fam Pract 2012;29:332-7.

4. R Core Team. R: A languange and environment for statistical computing. Vol. Vienna, Austria: R Foundation for Statistical Computing, 2015.

5. van Hassel D, Korevaar J, Batenburg R, Schellevis F. De toekomstvisie huisartsenzorg 2011, waar staat de huisartsenzorg anno 2014? , Vol. 1. Utrecht: NIVEL, 2015:1-85.

6. Martin TN, Groenning BA, Murray HM, Steedman T, Foster JE, Elliot AT, et al. St-segment deviation analysis of the admission 12-lead electrocardiogram as an aid to early diagnosis of acute myocardial infarction with a cardiac magnetic resonance imaging gold standard. J Am Coll Cardiol 2007;50:1021-8.

7. Kip MMA, Noltes AM, Koffijberg H, IJzerman MJ, Kusters R. Improving early exclusion of acute coronary syndrome in primary care: the added value of point-of-care troponin as stated by general practitioners. Manuscript submitted**,** 2016.

8. Nilsson S, Ortoft K, Molstad S. The accuracy of general practitioners' clinical assessment of chest pain patients. Eur J Gen Pract 2008;14:50-5.

9. Diercks DB, Peacock WFt, Hollander JE, Singer AJ, Birkhahn R, Shapiro N, et al. Diagnostic accuracy of a point-of-care troponin i assay for acute myocardial infarction within 3 hours after presentation in early presenters to the emergency department with chest pain. Am Heart J 2012;163:74-80 e4.

10. Granger CB, Goldberg RJ, Dabbous O, Pieper KS, Eagle KA, Cannon CP, et al. Predictors of hospital mortality in the global registry of acute coronary events. Arch Intern Med 2003;163:2345-53.

11. Pope JH, Aufderheide TP, Ruthazer R, Woolard RH, Feldman JA, Beshansky JR, et al. Missed diagnoses of acute cardiac ischemia in the emergency department. N Engl J Med 2000;342:1163-70.

12. Nallamothu B, Fox KA, Kennelly BM, Van de Werf F, Gore JM, Steg PG, et al. Relationship of treatment delays and mortality in patients undergoing fibrinolysis and primary percutaneous coronary intervention. The global registry of acute coronary events. Heart 2007;93:1552-5.

13. Bruins Slot MH, Rutten FH, van der Heijden GJ, Doevendans PA, Mast EG, Bredero AC, et al. Diagnostic value of a heart-type fatty acid-binding protein (h-fabp) bedside test in suspected acute coronary syndrome in primary care. Int J Cardiol 2013;168:1485-9.

14. Goldberg RJ, Currie K, White K, Brieger D, Steg PG, Goodman SG, et al. Six-month outcomes in a multinational registry of patients hospitalized with an acute coronary syndrome (the global registry of acute coronary events [grace]). Am J Cardiol 2004;93:288-93.

15. Sequist TD, Bates DW, Cook EF, Lampert S, Schaefer M, Wright J, et al. Prediction of missed myocardial infarction among symptomatic outpatients without coronary heart disease. Am Heart J 2005;149:74-81.

16. Mourad G, Alwin J, Stromberg A, Jaarsma T. Societal costs of non-cardiac chest pain compared with ischemic heart disease--a longitudinal study. BMC Health Serv Res 2013;13:403.

17. Ghushchyan V, Nair KV, Page RL, 2nd. Indirect and direct costs of acute coronary syndromes with comorbid atrial fibrillation, heart failure, or both. Vasc Health Risk Manag 2015;11:25-34.

18. Hakkaart-van Roijen L, van der Linden N, Bouwmans C, Kanters T, Tan SS. Bijlage 1. Kostenhandleiding: Methodologie van kostenonderzoek en referentieprijzen voor economische evaluaties in de gezondheidszorg., Vol. Rotterdam: Institute for Medical Technology Assessment, Erasmus Universiteit Rotterdam, 2015:1-120.

19. Goodacre S, Thokala P, Carroll C, Stevens JW, Leaviss J, Al Khalaf M, et al. Systematic review, meta-analysis and economic modelling of diagnostic strategies for suspected acute coronary syndrome. Health Technol Assess 2013;17:v-vi, 1-188.

20. Polanczyk CA, Kuntz KM, Sacks DB, Johnson PA, Lee TH. Emergency department triage strategies for acute chest pain using creatine kinase-mb and troponin i assays: A cost-effectiveness analysis. Ann Intern Med 1999;131:909-18.

21. Ward S, Lloyd Jones M, Pandor A, Holmes M, Ara R, Ryan A, et al. A systematic review and economic evaluation of statins for the prevention of coronary events. Health Technol Assess 2007;11:1-160, iii-iv.

22. Sullivan PW, Ghushchyan V. Preference-based eq-5d index scores for chronic conditions in the united states. Med Decis Making 2006;26:410-20.

23. Alter DA, Ko DT, Tu JV, Stukel TA, Lee DS, Laupacis A, et al. The average lifespan of patients discharged from hospital with heart failure. J Gen Intern Med 2012;27:1171-9.

24. Nederlandse Zorgautoriteit. Bijlage 1 bij tariefbeschikking tb/cu-7089-02: Tarievenlijst huisartsenzorg en multidisciplinaire zorg. 2015.

25. Nederlandse Zorgautoriteit. Open data van de nederlandse zorgautoriteit. <http://www.opendisdata.nl/> (Accessed March 29 2016).

26. Volksgezondheidenzorg.info. Cijfertool kosten van ziekten. https://kostenvanziektentool.volksgezondheidenzorg.info/tool/nederlands/?ref=kvz_v2l1b1p4r3c0i0t1j0o3y6a-1g0d207s54f0z0w2 (Accessed November 20 2015).

27. Rutten FH, Poos MJJC, Engelfriet PM. Hoe vaak komen hartfalen voor en hoeveel mensen sterven eraan? In: Volksgezondheid en toekomst verkenning. <http://www.nationaalkompas.nl/gezondheid-en-ziekte/ziekten-en-aandoeningen/hartvaatstelsel/hartfalen/omvang/> (Accessed March 31 2016).
