# Supplementary material for: The cost-utility of point-of-care troponin testing to diagnose acute coronary syndrome in primary care
Source: BMC Cardiovasc Disord. 2017 Aug 2;17:213. doi: 10.1186/s12872-017-0647-6 (PMC5541723; doi:10.1186/s12872-017-0647-6)
Supplement: Supplementary file 2 — Overview of model assumptions. This file contains an overview of all assumptions that have been used in the health economic analysis (DOCX 18 kb) [file 12872_2017_647_MOESM2_ESM.docx]

**Additional file 2: overview of model assumptions**

**Assumptions related to the diagnostic work-up of the general practitioner (GP):**

1. In case the GP perceives a patient to be at high risk of acute coronary syndrome (ACS), the patient is immediately referred to the hospital, without performing an electrocardiogram (ECG) or a point-of-care (POC) troponin test.
2. In case a GP has the availability of an ECG in his or her general practice, the GP is also assumed to perform an ECG in all chest pain patients presenting in their general practice, except for patients that are immediately referred to the hospital because the GP perceives a high probability of ACS in those patients.
3. In case the GP diagnoses a ST elevation myocardial infarction (STEMI) based on the ECG, the patient is immediately referred to the hospital, without performing a POC troponin test.
4. It is assumed that the POC troponin test is not used in patients presenting within four hours after symptom onset.
5. All patients that are referred to the hospital are assumed to be transported by ambulance.
6. In case the initial decision of the GP was to refer the patient, but the POC troponin level was negative, the probability that the GP would no longer refer this patient was 81%. For a positive POC troponin level in a patient that was initially not referred, this probability was found to be 83% [manuscript submitted, [1](#_ENREF_1)].
7. As a previous study indicates that 10 minutes is considered the maximum acceptable test duration for POC troponin [manuscript submitted, [1](#_ENREF_1)], and because literature has shown that POC troponin tests who meet this requirement are currently available [[2](#_ENREF_2)] it was assumed that the POC troponin test takes 10 minutes to perform.
8. The costs of performing a POC troponin test by the GP are assumed to be €15.

**Assumptions related to treatment as well as treatment outcomes:**

1. In case an ACS patient is not immediately referred to the hospital, it is assumed that the patient will present to the hospital at a later point in time. However, this leads to a delay in treatment, and consequently to an increased risk of mortality and developing heart failure.
2. A higher mortality rate per 10 minutes treatment delay has been reported for STEMI patients, indicating a slightly higher mortality rate attributable to the time delay caused by POC troponin testing. It is conservatively assumed that this time delay is also applicable to non-ST elevation myocardial infarction (NSTEMI) patients [[3](#_ENREF_3)].

**Assumptions related to productivity loss:**

1. All patients are assumed to retire at the age of 65, indicating that no costs of productivity loss are assumed to occur in patients aged 65 years and older.
2. As the distance to the GP is approximately 1.1 kilometer [[4](#_ENREF_4)], the travel time to the GP is set at 15 minutes. Therefore, a GP consultation is assumed to take 2 x 15 minutes travel time, plus 1 x 10 minutes consultation time = 40 minutes. In case a POC troponin test is performed, an additional time of 10 minutes is assumed.
3. The duration of an emergency department referral for chest pain is estimated by various hospitals to differ between 2 and 24 hours, but 8 hours is often considered as the average duration. Therefore, the duration of an ED referral, including the consultation at the GP, is estimated to take one working day, i.e. 8 hours.

**References**:

1. Kip, M.M.A., et al., Improving early exclusion of acute coronary syndrome in primary care: the added value of point-of-care troponin as stated by general practitioners, manuscript submitted, 2016.

2. Yang, Z. and D. Min Zhou, *Cardiac markers and their point-of-care testing for diagnosis of acute myocardial infarction.* Clin Biochem, 2006. **39**(8): p. 771-80.

3. Nallamothu, B., et al., *Relationship of treatment delays and mortality in patients undergoing fibrinolysis and primary percutaneous coronary intervention. The Global Registry of Acute Coronary Events.* Heart, 2007. **93**(12): p. 1552-5.

4. Hakkaart-van Roijen, L., et al., *Bijlage 1. Kostenhandleiding: methodologie van kostenonderzoek en referentieprijzen voor economische evaluaties in de gezondheidszorg.*, 2015, Institute for Medical Technology Assessment, Erasmus Universiteit Rotterdam: Rotterdam. p. 1-120.
