# Supplementary material for: The cost-utility of point-of-care troponin testing to diagnose acute coronary syndrome in primary care
Source: BMC Cardiovasc Disord. 2017 Aug 2;17:213. doi: 10.1186/s12872-017-0647-6 (PMC5541723; doi:10.1186/s12872-017-0647-6)
Supplement: Supplementary file 3 — Results of partial perfect value of information analysis. This file contains an overview of the results of the partial perfect value of information analysis (DOCX 13 kb) [file 12872_2017_647_MOESM3_ESM.docx]

**Additional file 3: results of partial perfect value of information analysis**

| **Parameter group:** | **Per person EVPPI (€)** | **Indexed to overall EVPI** | **EVPPI for the Netherlands per year (€)** |
| --- | --- | --- | --- |
| **Probabilities regarding population parameters** (age, gender, probabilities regarding STEMI, NSTEMI, UAP, death, heart failure, and the increased probability of mortality due to aging, missed ACS, and attributable to the time of POC troponin testing). | 0.00 | 0.00 | 147.18 |
| **Probabilities regarding POC testing and decision-making** (time to presentation, probability ECG, sensitivity of GP and POC troponin test, probability of revising referral decision). | 0.00 | 0.01 | 350.06 |
| **QALYs** (concerns all quality-of-life estimated used in the model). | 0.00 | 0.01 | 340.62 |
| **Productivity loss (in hours)** (productivity loss attributable to GP and hospital visits, as well as due to UAP, NSTEMI, STEMI, heart failure, and mortality). | 0.00 | 0.00 | 0.00 |
| **Cost parameters** (concerns all cost parameters used in the model). | 0.01 | 0.02 | 942.37 |
